# The Role of Methyl Canthin-6-one-2-carboxylate in Targeting the NLRP3 Inflammasome in Rheumatoid Arthritis Treatment

**DOI:** 10.3390/cimb47040254

**Published:** 2025-04-07

**Authors:** Chung-Che Tsai, Tin-Yi Chu, Po-Chih Hsu, Chan-Yen Kuo

**Affiliations:** 1 Department of Research, Taipei Tzu Chi Hospital, Buddhist Tzu Chi Medical Foundation, New Taipei City 231, Taiwan; chungche.tsai@gmail.com (C.-C.T.); tintin4125@gmail.com (T.-Y.C.); 2 Department of Dentistry, Taipei Tzu Chi Hospital, Buddhist Tzu Chi Medical Foundation, New Taipei City 231, Taiwan; 3 Institute of Oral Medicine and Materials, College of Medicine, Tzu Chi University, Hualien 970, Taiwan

**Keywords:** rheumatoid arthritis, NLRP3 inflammasome, methyl canthin-6-one-2-carboxylate, alternative medicine, traditional Chinese medicine

## Abstract

Rheumatoid arthritis (RA) is a chronic autoimmune disorder characterized by persistent synovial inflammation, joint destruction, and systemic complications. The nucleotide-binding domain, leucine-rich repeat family, pyrin domain-containing-3 (NLRP3) inflammasome plays a pivotal role in RA pathogenesis by driving the release of pro-inflammatory cytokines and exacerbating oxidative stress. Recent studies identified methyl canthin-6-one-2-carboxylate (Cant) as a potential therapeutic agent that modulates the NLRP3 inflammasome pathway. This review explores the mechanistic role of Cant in RA treatment, particularly its effect on oxidative stress, synovial macrophages, and inflammatory signaling pathways. Additionally, we discuss alternative and complementary approaches, such as gut microbiota modulation and mesenchymal stem cell-based therapies, in the management of RA. Although preliminary findings suggest that Cant exhibits promising anti-inflammatory effects, further preclinical and clinical studies are necessary to validate its therapeutic efficacy. Future research should focus on optimizing dosage, exploring combination therapies, and elucidating the broader implications of targeting the NLRP3 inflammasome for RA treatment.

## 1. Introduction

Recently, Current Issues in Molecular Biology featured a pivotal study by Chen et al., which investigated the therapeutic potential of methyl canthin-6-one-2-carboxylate (Cant) in the management of rheumatoid arthritis (RA) [1]. However, Cant is not commercially available and must be synthesized in the laboratory [2]. This autoimmune condition, characterized by chronic joint inflammation and progressive tissue damage, presents challenges for effective and safe therapeutic strategies [3]. A recent study by Chen et al. not only sheds light on the molecular mechanism of action of Cant but also highlights its role in modulating the inflammatory microenvironment via the nucleotide-binding domain, leucine-rich repeat family, pyrin domain-containing-3 (NLRP3) inflammasome pathway [1]. In addition, a key hallmark of RA pathology is the aberrant activation of the NLRP3 inflammasome in synovial macrophages, leading to the production of pro-inflammatory cytokines and increased oxidative stress [4]. Microbial pathogens further exacerbate this process by inducing ROS production, which drives NLRP3 inflammasome activation and intensifies inflammatory responses in RA [5]. In the present review, a novel therapeutic approach involving the identification of nuclear factor erythroid-2-related factor 2 (Nrf2), a key regulator of cellular redox homeostasis [6], as a target of Cant is proposed. Specifically, this study demonstrates that Cant attenuates reactive oxygen species (ROS)-mediated NLRP3 activation, thereby suppressing downstream inflammation. The noteworthy aspects of this study include the integration of network pharmacology and rigorous in vitro experimentation. By combining computational and biological approaches, the authors were able to elucidate the multi-target effects of Cant on pathways such as nuclear factor kappa-light-chain-enhancer of activated B cells (NF-κB) and mitogen-activated protein kinase (MAPK), which are central to RA pathogenesis [7,8]. The findings of this study align with the growing body of evidence supporting therapeutic modulation of the NLRP3 inflammasome in autoimmune diseases. Despite these compelling results, the authors highlight the need for further investigation. Although these data suggest the promising role of Cant in mitigating RA-related inflammation, its efficacy and safety must be validated in preclinical animal models and, eventually, in clinical trials. Exploring the effects of Cant on other cell types in the synovial microenvironment may also broaden our understanding of its therapeutic potential.

RA affects millions of individuals worldwide and causes severe joint dysfunction and systemic complications [9]. Current treatment strategies primarily involve disease-modifying antirheumatic drugs (DMARDs) and biologics that target inflammatory cytokines [10]. However, challenges, such as incomplete response rates and adverse effects, necessitate the development of novel therapeutic agents. The NLRP3 inflammasome has emerged as a promising target in RA treatment owing to its role in amplifying inflammation through interleukin (IL)-1β and IL-18 production [11]. This review highlights the potential of Cant in modulating this pathway and its broader implications for RA therapy.

## 2. Mechanism of Action of Methyl Canthin-6-one-2-carboxylate and Its Derivatives in RA and Other Diseases

An indole alkaloid derivative, Cant, was identified in a recent study [12]. This compound is found in plants such as *Zanthoxylum chiloperone*, *Aerva lanata*, *Eurycoma longifolia* roots, and *Simaba ferruginea* A. St.-Hil. [12]. Ding et al. reported that canthin-6-one derivatives have significant anti-proliferative effects on HT29 colon cancer cells [7]. Cant inhibits the pro-inflammatory functions of RA fibroblast-like synoviocytes (FLS), including migration, invasion, and the release of matrix metalloproteinases (MMPs) and inflammatory cytokines. This suppressive role of Cant is achieved by inhibiting the Hippo/YAP signaling pathway [12]. O’Donnell and Gibbons investigated the antibacterial properties of compounds isolated from a Mediterranean herb named *Allium neapolitanum* and identified two canthin-6-one alkaloids: canthin-6-one and 8-hydroxy-canthin-6-one. These compounds exhibited significant antibacterial activity, with minimum inhibitory concentrations ranging from 8 to 32 µg/mL against various fast-growing *Mycobacterium* species and from 8 to 64 µg/mL against multidrug-resistant and methicillin-resistant *Staphylococcus aureus* strains. This was the first study to report the presence of canthin-6-one alkaloids in the *Alliaceae* family and highlight their potential as antibacterial agents against resistant bacterial strains [13]. Taken together, these results suggest broader applicability to various diseases. Further studies are warranted to explore the therapeutic potential and clinical viability of these compounds.

## 3. The Role of the NLRP3 Inflammasome in RA Pathogenesis

**Figure 1:**
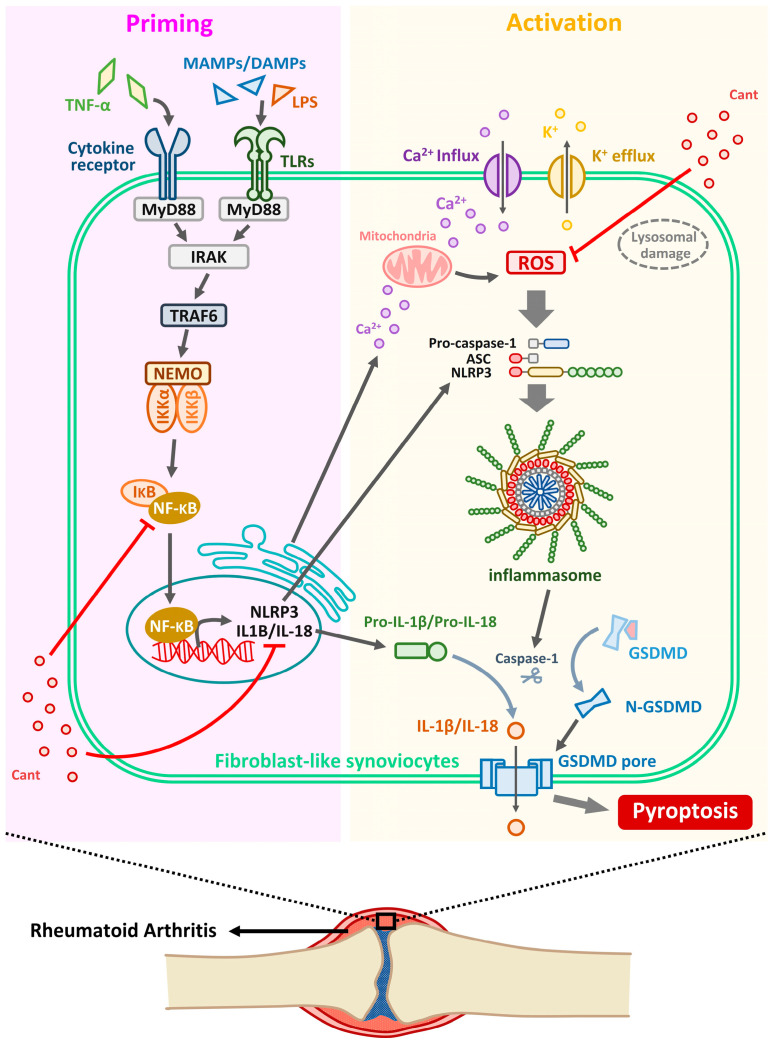
The activation mechanism of the NLRP3 inflammasome and the regulatory effects of Cant in RA. NLRP3 inflammasome activation occurs through two processes. In the priming step, activation of TLRs or cytokine receptors by MAMPs, DAMPs, or TNF-α stimulates downstream MyD88/IRAK/TRAF6/NF-κB signaling. The translocation of activated NF-κB into the nucleus promotes the expression of NLRP3, pro-IL-1β, and pro-IL-18. In the activation step, elevated Ca^2+^ levels, lysosomal damage, and ROS caused by mitochondrial dysfunction, along with K^+^ efflux leading to a reduced intracellular K^+^ level, trigger inflammasome assembly. These intracellular events facilitate the formation of the inflammasome complex, consisting of NLRP3, ASC, and pro-caspase-1. The inflammasome then catalyzes the conversion of pro-caspase-1 into active caspase-1. Activated caspase-1 subsequently cleaves pro-IL-1β and pro-IL-18 into their active forms. Additionally, it cleaves the GSDMD protein, initiating the oligomerization of N-GSDMD to form membrane pores. This process promotes the secretion of IL-1β and IL-18 and further induces pyroptosis. In addition, the inhibitory effect of Cant on NLRP3 inflammasome formation involves the suppression of NF-κB activation, downregulation of NLRP3, pro-IL-1β, and pro-IL-18 expression, and attenuation of ROS production. MAMP, microbe-associated molecular patterns; DAMPs, damage-associated molecular patterns; LPS, lipopolysaccharide; MyD88, myeloid differentiation primary response 88; IRAK, interleukin-1 receptor-associated kinase; TRAF6, TNF receptor-associated factor 6; IKK, IκB kinase.

The NLRP3 inflammasome plays a critical role in RA pathogenesis [14]. Its activation drives the formation of inflammasomes, a hallmark of pyroptosis—a form of programmed cell death marked by cell swelling, membrane rupture, and the release of pro-inflammatory cytokines [15]. By contrast, necrosis is an unregulated form of cell death resulting from acute injury, leading to uncontrolled cell lysis and inflammation. Another distinct form, apoptosis, is a tightly regulated, non-inflammatory process characterized by cell shrinkage, DNA fragmentation, and membrane blebbing, essential for maintaining tissue homeostasis [15]. The present review summarizes the activation of NLRP3 inflammasome involved in RA signaling (Figure 1). First, priming signals, including toll-like receptors (TLRs) and NF-κB signaling, upregulate NLRP3 and pro-IL-1β transcription in response to damage-associated molecular patterns and pathogen-associated molecular patterns [16]. Second, the induction of NLRP3 oligomerization (activation signal) by mitochondrial dysfunction, oxidative stress, and ionic flux (K^+^ efflux and Ca^2+^ influx) results in the activation of caspase-1, which processes IL-1β and IL-18 into their active forms [17]. Upon activation, NLRP3 recruits apoptosis-associated speck-like protein containing CARD and procaspase-1, forming an active inflammasome complex that cleaves pro-inflammatory cytokines and induces pyroptotic cell death [18].

The NLRP3 inflammasome is a multiprotein complex that senses cellular stress signals and triggers caspase-1 activation, leading to the maturation of pro-inflammatory cytokines [19]. In RA, excessive NLRP3 activation contributes to synovial hyperplasia, increased immune cell infiltration, and cartilage destruction [20]. Additionally, oxidative stress exacerbates inflammation by promoting ROS-mediated activation of NLRP3, thereby initiating a vicious cycle of tissue damage [21]. As shown in Figure 1, Cant exerts its inhibitory effect on NLRP3 inflammasome formation in RA by suppressing NF-κB activation, downregulating NLRP3, pro-IL-1β, and pro-IL-18 expression, and reducing ROS production, thereby preventing pyroptosis.

Numerous clinical and epidemiological studies have demonstrated a strong association between RA and periodontal disease (PD) [22,23]. *Porphyromonas gingivalis*, a key periodontal pathogen, produces peptidyl arginine deiminase (PAD), an enzyme that catalyzes protein citrullination [24]. This process is believed to trigger the formation of anti-citrullinated protein antibodies (ACPAs), a hallmark of RA pathogenesis [25,26,27]. Furthermore, elevated levels of anti-cyclic citrullinated peptide (CCP) antibodies have been positively correlated with RA in animal models [23]. Citrullinated proteins generated via PAD may also promote NETosis by regulating NLRP3 inflammasome activation, further contributing to RA inflammation [28]. Collectively, these findings suggest that *P. gingivalis*-induced biomarkers are closely linked to RA pathogenesis.

## 4. Potential Benefits of Traditional and Alternative Medicine in RA Treatment

RA remains a challenging autoimmune disease, requiring effective and safe treatments. Traditional treatments include nonsteroidal anti-inflammatory drugs, DMARDs, glucocorticoids, and biological agents; however, the long-term use of these medications can lead to adverse side effects [29,30]. Traditional Chinese medicine (TCM) and alternative medicine are considered potentially beneficial for RA treatment [31]. The potential TCMs that inhibit the physiological effects of RA are listed in Table 1. In TCM, RA is believed to result from the invasion of “wind, cold, and dampness”, and herbs that dispel these factors are used to alleviate pain [29]. Biqi capsules, in combination with methotrexate, could be a promising alternative treatment for RA as it acts via regulation of the inflammatory response derived from Th2, offering similar efficacy with a better safety profile compared to the standard regimen involving leflunomide combined with methotrexate [32], consistent with the findings of a systematic review and meta-analysis conducted by Chen et al. [33]. According to a nationwide population-based study conducted in Taiwan, TCM use was highly prevalent among patients with RA, with specific usage patterns. The most prescribed herbal formulas include Shang-Jong-Shiah-Tong-Yong-Tong-Feng-Wan and *Rhizoma Corydalis* [34]. The main components of traditional herbal medicines thought to ameliorate RA are flavonoids, phenolic acids, alkaloids, and triterpenes [35]. Numerous bioactive compounds found in herbal products have shown therapeutic potential, many of which have been developed into drugs used worldwide to treat inflammatory and autoimmune conditions [36]. As with conventional medicine, East Asian herbal medicine has also been reported to reduce inflammatory pain associated with RA according to 11 databases containing English, Korean, Chinese, and Japanese literature and randomized controlled trials comparing East Asian herbal medicine to conventional medicine [37]. Moreover, a systematic review evaluated the efficacy and safety of combining conventional DMARDs with Chinese herbal medicines to treat RA. The results showed that the most common Chinese herbal medicines, including *Angelicae Sinensis* Radix, *Paeoniae Radix* Alba, *Cinnamomi Ramulus*, *Glycyrrhizae Radix* et Rhizoma, and *Clematidis Radix* et Rhizoma, demonstrated pharmacological benefits that contributed to better RA outcomes [38]. Ahmed et al. investigated the anti-arthritic potential of *Parmotrema tinctorum*, a lichen species, in rats with arthritis induced by complete Freund’s adjuvant [39]. Three herbal extracts, namely, modified Huo-luo-xiao-ling dan, *Celastrus aculeatus* Merr., the polyphenolic fraction of green tea (*Camellia sinensis*), and celastrol, a bioactive component of *Celastrus*, are reviewed here, considering our systematic studies [40,41]. Tong and Moudgil investigated the effects of an ethanolic extract of *Celastrus aculeatus* on autoimmune arthritis using a rat model of adjuvant arthritis, which closely resembles human RA. The authors found that administering this extract both before and after disease onset significantly reduced arthritis severity via regulation of heat-shock protein 65 (Hsp65). Specifically, the extract decreased T-cell proliferation and altered cytokine production in response to Hsp65, suggesting that *Celastrus aculeatus* may exert its anti-arthritic effects by modulating antigen-specific immune responses [42]. Specific catechins, particularly those containing a gallate ester extracted from *Camellia sinensis*, effectively inhibit the breakdown of proteoglycans and type II collagen in both bovine and human cartilage samples. Therefore, certain green tea catechins possess chondroprotective properties, indicating that green tea consumption may help to prevent or slow the progression of arthritis by reducing inflammation and cartilage degradation [43]. Natural products, particularly those with antioxidant properties, may also provide additional therapeutic benefits [44]. Gamma-linolenic acid is a polyunsaturated fatty acid found in human milk and several botanical seed oils, such as blackcurrant and evening primrose, and is typically consumed as a dietary supplement. It has been shown that the gamma-linolenic acid in some herbal medicines can improve pain, tender joints, and stiffness in patients with RA; however, there is only a moderate degree of evidentiary support for this [45,46]. Several clinical trials have incorporated systemic RA-associated biomarkers, such as ACPAs, rheumatoid factor (RF), and C-reactive protein (CRP), to evaluate the immunological and clinical efficacy of various therapeutic strategies. These biomarkers serve not only as diagnostic tools but also as indicators of disease activity and treatment response. Biologic agents targeting pro-inflammatory pathways, including tumor necrosis factor-α (TNF-α), IL-6, and B cells, have been extensively studied in this context. For instance, treatment with tocilizumab has been shown to significantly reduce CRP levels due to its direct inhibition of IL-6 signaling, a key driver of hepatic acute-phase reactants [47]. Rituximab, a B cell-depleting agent, has demonstrated variable but occasionally significant reductions in ACPA and RF titers in patients with established RA [48]. Similarly, TNF inhibitors such as adalimumab and etanercept have been associated with decreased CRP and, in some cases, modest changes in autoantibody profiles over time [49]. Collectively, these findings underscore the utility of systemic RA-associated biomarkers in clinical trials, both for monitoring therapeutic response and for enhancing precision medicine approaches in RA management. However, further rigorous clinical studies are necessary to validate these findings and optimize integrative treatment approaches for RA management.

## 5. Role of the Gut Microbiota in RA: Mechanisms, Therapeutic Potential, and Clinical Implications

The gut microbiota plays a very important role in RA pathogenesis through mechanisms that include the production of pro-inflammatory metabolites, impaired intestinal mucosal barrier function, and molecular mimicry of autoantigens [64,65,66]. Patients with RA exhibit significant alterations in their intestinal microbiota compared to healthy individuals [67]. Various strains of *Lactobacillus* and *Bifidobacteria*, particularly *Lactobacillus casei*, have demonstrated the potential to reduce RA disease activity [68]. Pang et al. reported that the therapeutic effects of *Atractylodes koreana* on RA may be attributed to its ability to downregulate inflammatory factors, including TNF-α, IL-1, IL-1β, IL-2, IL-6, and high-sensitivity CRP, as well as restore balance to the gut microbiota and short-chain fatty acids in rats models [69]. A previous study highlighted the potential of targeting the gut microbiota as an effective therapy for RA. Probiotics and prebiotics have demonstrated their ability to modulate the gut microbiota, reduce inflammation, and alleviate RA symptoms in both animal models and patients. Despite the challenges in probiotic research, such as identifying specific targets and mechanisms, their health benefits continue to drive further exploration. Diet plays a crucial role in shaping the gut microbiota and influences the development of RA. Additionally, fecal microbiota transplantation has shown preliminary efficacy in a single patient. These different interventions may, in turn, affect systemic immune responses, including RA-related biomarkers such as anti-CCP antibodies, pro-inflammatory cytokines (e.g., IL-6, TNF-α), and CRP levels [70,71,72]. However, larger clinical trials are necessary to confirm its safety and effectiveness [73]. By contrast, a recent study concluded that conflicting reports on *Prevotellaceae* overabundance in RA may result from sampling within a heterogeneous population along a dynamic disease spectrum, and that gut microbiome alterations may manifest in later stages, i.e., during the transition to clinical arthritis [74]. Similar results showed that the presence of the genera *Subdoligranulum* and *Fusicatenibacter* was associated with favorable responses to second-line conventional synthetic DMARDs in patients who did not adequately respond to the initial therapy [75]. The role of the gut microbiota in the pathogeneses of osteoarthritis, RA, and spondylarthritis involves the “gut–joint axis”. Gut dysbiosis contributes to systemic inflammation, leading to joint diseases through mechanisms such as increased intestinal permeability, molecular mimicry, and immune system activation [76,77]. In conclusion, growing evidence underscores the significant role of the gut microbiota in RA pathogenesis, highlighting mechanisms such as pro-inflammatory metabolite production, impaired intestinal barrier function, and molecular mimicry. Various probiotics, including *Lactobacillus casei* and *Bifidobacteria* strains, have shown potential in reducing RA activity [78], whereas herbal treatments, such as *Atractylodes koreana*, demonstrate anti-inflammatory effects and microbiota modulation [79]. In pre-clinical RA cohorts, particularly among individuals who are seropositive but asymptomatic, alterations in the gut microbiome may modulate disease risk or delay clinical onset. Several studies have demonstrated associations between specific microbial taxa—such as *Prevotella copri*—and increased RA risk, suggesting that gut microbial shifts may be reflected in risk assessment and disease prediction models [65,80,81]. Despite the challenges in probiotic research, targeting the gut microbiota remains a promising approach for RA therapy, with dietary interventions and fecal microbiota transplantation showing great potential [66,82]. Further research, including large-scale clinical trials, is necessary to validate these findings and develop microbiota-targeted therapeutic strategies.

## 6. Mesenchymal Stem/Stromal Cells as a Potential Therapy for RA

Mesenchymal stem cells (MSCs) can differentiate into various cell types, including adipocytes, chondrocytes, and osteoblasts [83]. They can also serve as a promising tool for stem cell-based therapies because of their unique immunostimulatory properties, providing new strategies for the treatment of RA in both experimental studies and clinical trials [84,85]. He et al. demonstrated that interferon-γ (IFN-γ) plays a crucial role in the effectiveness of human umbilical cord mesenchymal stem (stromal) cell transplantation for RA treatment and that combining MSCs with IFN-γ can significantly enhance the clinical outcomes of MSC-based therapy in patients with active RA [86]. The results of a non-randomized, open-label phase I/IIa evaluation of the safety and efficacy of intravenous infusion of autologous adipose-derived MSCs among patients with active RA demonstrated significant improvements in joint function, as indicated by reductions in swollen and tender joint counts. While levels of inflammatory markers such as TNF-α, IL-6, and the erythrocyte sedimentation rate remained unchanged, a modest improvement in CRP levels was observed [87]. A previous study investigated the immunomodulatory effects of MSCs and their extracellular vesicles (EVs) on RA CD4^+^ T cells and FLS. EVs derived from IFN-β-primed MSCs were more effective than naïve MSC-EVs or MSCs alone in suppressing key RA-associated cytokines, reducing CD4^+^ T-cell polyfunctionality, and restoring T-regulatory cell frequency. Additionally, IFN-β-primed MSC-EVs significantly inhibited RA FLS migration and downregulated surface markers CD34 and HLA-DR. These findings suggest that IFN-β-primed MSC-EVs have strong therapeutic potential for managing RA and act by modulating immune responses and fibroblast activity [88]. Although the efficacy of MSC therapy for RA remains inconclusive, the data indicate a potential trend toward clinical benefits. These findings suggest that MSC therapy could be considered an alternative treatment for RA patients who do not respond to conventional synthetic DMARDs and for whom biological DMARDs are not viable options. Importantly, MSC therapy has demonstrated a favorable safety profile with no reported life-threatening events. However, owing to methodological limitations and significant heterogeneity among studies, further high-quality, large-scale randomized controlled trials with extended follow-up periods are necessary to validate these preliminary findings and establish the clinical efficacy of MSC therapy against RA [89]. It has been reported that RA MSCs isolated from the bone marrow exhibited reduced proliferative activity and abnormal migration but showed no significant differences in cytokine profiles compared to controls [90]. Although RA MSCs retain their ability to suppress peripheral blood mononuclear cell proliferation, decrease the number of Tfh cells, and induce Treg cell polarization, they have an impaired capacity to inhibit Th17 cell polarization. This deficiency has been linked to reduced CCL2 expression in RA MSCs following their interaction with CD4^+^ T cells [90]. Moreover, according to a meta-analysis, MSCs have consistently demonstrated therapeutic benefits in preclinical studies with animal models of RA based on clinical scores, histological scores, and paw thickness. Despite correction for publication bias, these findings remained robust [91]. Taken together, MSCs have shown a favorable safety profile and potential clinical benefits for RA treatment [92]. However, further research is essential to fully understand their impact on the dysregulated proinflammatory environment in patients with RA. Although MSCs may serve as a promising option for severe RA cases that are unresponsive to conventional therapies, long-term follow-up and standardized treatment protocols remain to be fully established, particularly with respect to repeated dosing, donor variability, and cell source (autologous vs. allogeneic). As such, continued longitudinal studies are required to thoroughly evaluate potential immunogenicity, tumorigenicity, and other unforeseen complications. Future studies should prioritize well-designed multicenter randomized clinical trials with sufficient sample sizes and carefully selected patient populations that meet RA diagnostic criteria to provide a robust assessment of the efficacy of MSC therapy [93]. However, from an economic perspective, MSC therapy is currently associated with high production and clinical application costs, largely due to complex protocols for cell isolation, expansion, quality assurance, and regulatory compliance. These costs far exceed those of conventional pharmacologic treatments. However, given the potential of MSCs to induce sustained remission or modify disease progression, there may be long-term cost-saving benefits by reducing the need for chronic immunosuppression and managing disease complications. Future cost-effectiveness analyses are essential to determine the feasibility of widespread clinical adoption of MSC-based therapies in RA.

## 7. Summary

This review explores the role of the NLRP3 inflammasome in RA and the potential therapeutic effects of Cant. It highlights how Cant modulates inflammatory pathways, reduces oxidative stress, and suppresses synovial macrophage activation in patients with RA. Mechanistic insights are provided through discussions on the NF-κB and MAPK signaling pathways, which are central to RA pathogenesis. This article also explores traditional and alternative medicinal approaches, including herbal treatments and gut microbiota modulation, as adjuncts to RA management. Additionally, the potential of mesenchymal stem/stromal cells as a novel RA therapy is evaluated. Although Cant shows promise in targeting the NLRP3 inflammasome and reducing inflammation, further research through preclinical and clinical trials is needed to establish its efficacy and safety.

## 8. Conclusions

Cant is a promising therapeutic candidate for RA, as it targets the NLRP3 inflammasome and mitigates oxidative stress-induced inflammation. By modulating key inflammatory pathways, such as the NF-κB, MAPK, and Hippo/YAP signaling pathways, Cant effectively suppresses synovial macrophage activation and FLS function, thereby reducing joint damage. Additionally, growing evidence highlights the interplay between the gut microbiota, mesenchymal stem cells, and alternative medicine in RA treatment, further emphasizing the need for integrative therapeutic strategies. Although preclinical studies indicate the potential efficacy of Cant, further investigations, including in vivo studies and clinical trials, are necessary to validate its safety, pharmacokinetics, and long-term effects in patients with RA. Future research should also explore its combined potential with existing DMARDs and biologics to enhance therapeutic outcomes. By bridging molecular insights into clinical applications, Cant may offer a novel and effective approach for RA management.

## Figures and Tables

**Table 1:** The potential of TCM to inhibit the related actions in RA.

TCM	Source	Mechanism	Related Actions in RA	References
Cant	*Zanthoxylum chiloperone*, *Aerva lanata*, *Eurycoma longifolia*, and *Simaba ferruginea* A. St.-Hil.	IL-1β, IL-6, IL-18, tumor necrosis factor-α (TNF-α), nitric oxide (NO), cyclooxygenase-2 (COX-2), and NLRP3	Anti-inflammatory	[1]
Biqi capsule (combined with methotrexate)	*Strychnos nux-vomica* L., *Pheretima aspergillum* (E. Perrier), *Codonopsis pilosula* (Franch.) Nannf., *Poria cocos* (Schw.) Wolf., *Atractylodes macrocephala* Koidz., *Ligusticum chuanxiong* Hort., *Salvia miltiorrhiza* Bunge, *Panax notoginseng* (Burk.) F. H. Chen ex C. Chow, *Achyranthes bidentata* BL., and *Glycyrrhiza uralensis* Fisch.	IL-4 and IL-13 signaling	Anti-inflammatory	[32,33]
Extract	*Parmotrema tinctorum*	TNF-α	Anti-inflammatory	[39]
Huo-luo-xiao-ling dan	*Angelica sinensis* (Oliv.) Diels, *Salvia miltiorrhiza* Bge., *Boswellia carterii* Birdw., and *Commiphora myrrha* Engl.	Chemokines, IL-6, IL-17, MMP-2, and MMP-9	Anti-inflammatory	[41]
Ethanol extract	*Celastrus aculeatus* Merr.	Interferon-γ (IFN-γ), IL-10, and NO production	Anti-inflammatory	[42]
Catechins	*Camellia sinensis*	TNF and IL-1	Anti-inflammatory and slowing cartilage breakdown	[43]
Gamma-linolenic acid	Plant seed oils	NF-κB, activator protein 1 (AP-1), and IL-1β	Anti-inflammatory	[45,46,50]
Glycyrol	*Glycyrrhiza uralensis*	NF-κB, nuclear factor of activated T cell (NFAT), and IL-2	Downregulated autoimmune reactions	[51]
Liquiritin	*Glycyrrhiza uralensis*	MAPK and caspase-3	Anti-inflammatory	[52]
Gingerol	*Zingiber officinale* Rosc.	IL-1 and IL-6 signaling	Anti-inflammatory	[53]
Ethyl acetate fraction	*Angelica sinensis*	IL-1β signaling	Proliferation of RA synovial fibroblasts	[54]
Total glucosides of peony	*Paeonia lactiflora* Pall.	Prostaglandin E2 (PGE2), TNF-α, IL-1β	Anti-inflammatory	[55]
Polysaccharide	*Saposhnikovia divaricata*	TNF-α, IL-1β, p53, and caspase-3	Anti-inflammatory	[56]
Cinnamic acid (combined with mangiferin)	*Cinnamomum cassia*	IL-1β, IL-18, TLR4, NF-κB, and NLRP3	Anti-inflammatory	[57,58]
Acidic polysaccharides	*Ephedra sinica*	TLR4 and MAPK signaling	Anti-inflammatory and immuno-suppressive	[59]
Atractylone	*Atractylodes macrocephala* Koidz	NO production	Anti-inflammatory	[60]
Senkyunolide A	*Ligusticum chuanxiong* Hort.	TNF-α and NF-κB	Anti-inflammatory	[61]
Polyacetylenes	*Notopterygium incisum* Ting	NO production	Anti-inflammatory	[62]
Triterpenoids	*Poria cocos Schw.* Wolf	NO, PGE2, and COX-2	Anti-inflammatory	[63]

## Data Availability

Not applicable.

## Abbreviations

The following abbreviations are used in this manuscript:

RA	rheumatoid arthritis
Cant	methyl canthin-6-one-2-carboxylate
NLRP3	nucleotide-binding domain, leucine-rich repeat family, pyrin domain-containing-3
ROS	reactive oxygen species
TCM	traditional Chinese medicine
DMARD	disease-modifying antirheumatic drug
NF-κB	nuclear factor kappa-light-chain-enhancer of activated B cells
MAPK	mitogen-activated protein kinase
IL	interleukin
FLS	fibroblast-like synoviocytes
MMP	matrix metalloproteinase
TNF-α	tumor necrosis factor-α
MSC	mesenchymal stem cell
EV	extracellular vesicles
CRP	C-reactive protein
RF	rheumatoid factor
ACPAs	anti-citrullinated protein antibodies
CCP	cyclic citrullinated peptide
PD	periodontal disease
PAD	peptidyl arginine deiminase
MAMPs	microbe-associated molecular patterns
DAMPs	damage-associated molecular patterns
LPS	lipopolysaccharide
MyD88	myeloid differentiation primary response 88
IRAK	interleukin-1 receptor-associated kinase
TRAF6	TNF receptor-associated factor 6
IKK	IκB kinase
